# New Clathrin-Based Nanoplatforms for Magnetic Resonance Imaging

**DOI:** 10.1371/journal.pone.0035821

**Published:** 2012-05-01

**Authors:** Gordana D. Vitaliano, Franco Vitaliano, Jose D. Rios, Perry F. Renshaw, Martin H. Teicher

**Affiliations:** 1 Laboratory of Developmental Psychopharmacology, Brain Imaging Center, Department of Psychiatry, Harvard Medical School, McLean Hospital, Belmont, Massachusetts, United States of America; 2 VXM, Boston, Massachusetts, United States of America; 3 The Brain Institute, University of Utah School of Medicine, Salt Lake City, Utah, United States of America; Stanford, United States of America

## Abstract

**Background:**

Magnetic Resonance Imaging (MRI) has high spatial resolution, but low sensitivity for visualization of molecular targets in the central nervous system (CNS). Our goal was to develop a new MRI method with the potential for non-invasive molecular brain imaging. We herein introduce new bio-nanotechnology approaches for designing CNS contrast media based on the ubiquitous clathrin cell protein.

**Methodology/Principal Findings:**

The first approach utilizes three-legged clathrin triskelia modified to carry 81 gadolinium chelates. The second approach uses clathrin cages self-assembled from triskelia and designed to carry 432 gadolinium chelates. Clathrin triskelia and cages were characterized by size, structure, protein concentration, and chelate and gadolinium contents. Relaxivity was evaluated at 0.47 T. A series of studies were conducted to ascertain whether fluorescent-tagged clathrin nanoplatforms could cross the blood brain barriers (BBB) unaided following intranasal, intravenous, and intraperitoneal routes of administration. Clathrin nanoparticles can be constituted as triskelia (18.5 nm in size), and as cages assembled from them (55 nm). The mean chelate: clathrin heavy chain molar ratio was 27.04±4.8: 1 for triskelia, and 4.2±1.04: 1 for cages. Triskelia had ionic relaxivity of 16 mM^−1^s^−1^, and molecular relaxivity of 1,166 mM^−1^s^−1^, while cages had ionic relaxivity of 81 mM^−1^s^−1^ and molecular relaxivity of 31,512 mM^−1^s^−1^. Thus, cages exhibited 20 times higher ionic relaxivity and 8,000-fold greater molecular relaxivity than gadopentetate dimeglumine. Clathrin nanoplatforms modified with fluorescent tags were able to cross or bypass the BBB without enhancements following intravenous, intraperitoneal and intranasal administration in rats.

**Conclusions/Significance:**

Use of clathrin triskelia and cages as carriers of CNS contrast media represents a new approach. This new biocompatible protein-based nanotechnology demonstrated suitable physicochemical properties to warrant further in vivo imaging and drug delivery studies. Significantly, both nanotransporters crossed and/or bypassed the BBB without enhancers. Thus, clathrin nanoplatforms could be an appealing alternative to existing CNS bio-nanotechnologies.

## Introduction

A major focus in contrast agent research has been on molecular-level imaging, encompassing the study of receptors, transporters, enzymes, genes and intracellular processes. Positron emission tomography (PET) is among the most sensitive molecular imaging techniques, especially for central nervous system (CNS) applications. However, PET is limited by low spatial resolution, the need for some radioactive tracers to be produced locally, and limited availability of these tools and techniques.

Magnetic Resonance Imaging (MRI) is a widely used noninvasive visualization technique with high spatial resolution, but low sensitivity for visualization of molecular targets [1]. To improve MRI sensitivity for brain imaging, several contrast agent (CA) nano-delivery strategies have been designed [2], [3], [4], [5]. For example, by attaching paramagnetic (e.g., gadolinium) or superparamagnetic (e.g., iron oxide) agents to macromolecules relaxivity of MRI contrast agents can be significantly improved providing useful tracers [6], [7], [8]. Attached antibodies or ligands can also provide selective targeting [8], [9]. Also, dual imaging nanoplatforms detectable both by MRI and fluorescent microscopy can be used to delineate small primary tumors and metastases [10].

Over the past 30 years, various protein-based nanoplatforms, dendrimers, nanogels and other polymeric nanoparticles, liposomes, micelles, solid-lipid nanoparticles and Fullerenes, to name some, have been developed that show promise for imaging and also for delivery of different CNS therapies [11], [12], [13], [14], [15], [16], [17], [18]. Each nanotechnology has its own strengths, but also respective weaknesses [19], [20]. Stability of nanoplatforms has also been a problem, but can be improved using several strategies [8]. However, each strategy poses its own risks [8].

Protein based nanoplatforms have shown great promise as CA carriers. For example, one of the first macromolecular CA, albumin-Gd-DTPA, exhibited molecular relaxivity of 273 mM^−1^s^−1^, and ionic relaxivity of 14 mM^−1^s^−1^ [21]. Although widely used in preclinical studies, the covalently bound albumin-Gd-DTPA complexes have not been applied clinically, because of their slow clearance. However, the most recent Gd-based blood pool agents (e.g., MS-325, Gd-BOPTA) non-covalently attach to human serum albumin (HSA), which significantly improves their relaxivity and their pharmacokinetic properties [22], [23], [24]. But, they cannot cross an intact BBB. Some proteins (e.g., antibodies) have excellent targeting abilities, but limited loading efficacy. Others, like viruses, have ultra-high relaxivity, but are highly immunogenic. Finding an appropriate nontoxic, non-immunogenic, efficient CA carrier that can also cross the BBB has been a real challenge.

Several transport mechanisms are known to be involved in uptake of elements by the brain across the BBB [5], [25], [26]. A noninvasive nano-delivery mechanism to the CNS would be highly desirable in clinical imaging, and for nano-medicine in general. Invasive and noninvasive methods have been developed to deliver various types of elements across the BBB, but their clinical effectiveness has not been shown to be better than existing therapeutic methods [2]. New BBB-passing technologies include: lipidization, chemical or mechanical alteration of the BBB, convection-enhanced delivery (CED), and active and/or facilitated transport.

With respect to BBB passing of CA nanoparticles, various technologies and methods can be used with varying degrees of success. These typically entail nanoparticle functionalization with different types of molecules, including: surfactants (e.g., polysorbate 80), anti-transferrin or insulin receptor antibodies, single domain antibodies, and different peptide vectors (e.g., SynB vectors, Penetratin and TAT) [27]. On the other hand, intranasal delivery provides a direct transport pathway for nanoparticles into the brain by bypassing the BBB, which also may be useful in imaging [28].

A new method has been developed for non-invasive delivery of CA into the CNS, which further has the potential to enable high-resolution imaging. This method utilizes clathrin protein, and in particular, uses clathrin triskelia (CTs) [29]. These three-legged proteins are found in human, animal, plant, and fungal cells [30], [31], [32]. Clathrin triskelia can self-assemble into clathrin cages (CCs) ranging from 30 nm to 100 nm in size. CCs can encapsulate lipid vesicles [33], resulting in clathrin-coated vesicles (CCVs). These are the primary intracellular delivery vehicles responsible for receptor-mediated endocytosis at the plasma membrane, and for sorting of proteins at the trans-Golgi network [30], [31], [32]. Clathrin-mediated endocytosis is important for efficacy of anti-receptor monoclonal antibody-based tumor therapy, and for susceptibility to double-strained RNA-mediated gene silencing [34]. CCVs have a native ability to simultaneously carry different types of elements, such as: antibodies, hormones, growth factors, and neurotransmitters [30]. The rigid clathrin protein cage stabilizes its cargo and environmentally sequesters the vesicle and its contents. The clathrin lattice is also durable, is about 100-fold stiffer than the typical liposome [35] and is resistant to pH changes and trypsin digestion [36]. It also has multiple groups that can easily be modified (e.g., lysine, cysteine). These manifold qualities make clathrin structures suitable for study as CA nano-transporters [29]. Clathrin is shown to be active outside cells, natively crosses cell membranes, moves between neurons [37], and is active at the BBB [38], [39], which further suggest new CNS imaging capabilities. Accordingly, we set out to find whether clathrin could be used in MRI to improve relaxivity of contrast agents for CNS imaging.

In this study, the goal was to develop nontoxic, self-assembled, clathrin-based nanoplatforms for imaging within the CNS. The first objective was to chelate clathrin protein for MRI, and determine chelate ligand 2-(4-Isothiocyanatobenzyl)-diethylene-triamine-pentaacetic acid (DTPA-ITC) to clathrin protein molar ratio (L/P). The second objective was to attach a metal ion often used in imaging (e.g., Gadolinium), and determine T_1_ relaxivity for MRI applications. The final objective was to attach a fluorescent tag (e.g., fluorescein-isothiocyanate (FITC) or rhodamine), and, by using fluorescent imaging, test if the clathrin nanoplatforms could cross or bypass the BBB in rats.

## Methods

### Ethics Statement

Experiments were conducted in accordance with National Institutes of Health 1996 Guide for the Care and Use of Laboratory Animals and approved by McLean Hospital’s Institutional Animal Care and Use Committee (Protocol #07-6/2-21).

### Animals

Male Sprague Dawley (SD) rats (250 g–300 g) (Charles River, Boston, MA) were housed with ad libitum food and water in constant temperature and humidity conditions on a 12 hr. light/dark cycle.

### Reagents

Unless otherwise indicated, all chemicals were from Sigma-Aldrich, and included: sodium azide, gadolinium chloride, yttrium chloride, arsenazo III, EDTA, Isocyanatobenzyl-DTPA (Macrocyclics, Dallas, TX), Maleimide-poly-(ethylene-glycol)-N-hydroxysuccinimide (JenKen Tech., Allen, TX), rhodamine 110, FITC Labeling Kit (Pierce, Rockford, IL).

The compositions of the buffers were as follows: a) triskelia “dissociation buffer”, Tris (tris(hydroxymethyl)-aminomethane) buffer, 0.5 M Tris-HCl, 3 mM dithiothreitol (DTT), the pH = 7; b) triskelia “chelation buffer”, HEPES [N-(2-Hydroxyethyl) piperazine-N’-ethanesulfonic acid] buffer, 0.1 M HEPES, the pH = 8.5; c) cages “assembly buffer”, MES [2-(N-morpholino) ethanesulfonicacid] buffer, 50 mM MES Na, 100 mM NaCl, 2 mM DTT, the pH = 6.5; d) cages “storage buffer”, MES buffer, 20 mM MES Na, 2 mM DTT, the pH = 6.2; e) phosphate buffer, 50 mM KH_2_P0_4_, the pH (6.7–8.3) was adjusted with the addition of 0.1 M NaOH; e) 0.1 M ammonium acetate buffer, the pH (5.5–6) and f) 0.15 M sodium acetate buffer, the pH = 4.

### Protein Isolation

Clathrin-coated vesicles were isolated from fresh rat livers and brains, and clathrin triskelia and adaptor proteins isolated using standard methods [33]. Clathrin triskelia (5 mg/ml) in 0.5 M Tris buffer (pH 7) was dialyzed against at least a 500-fold volume excess of 0.1 M HEPES buffer (pH 8.5) containing 50 mM EDTA for 8 hours at 4°C and protein concentration determined by Bradford protein assay (Bio-Rad, Hercules, CA).

### Determination of the Isothiocyanatobenzyl-DTPA to Protein Molar Ratio

#### Triskelia

A 120-fold molar excess of 2-(4-Isothiocyanatobenzyl)-diethylene-triamine-pentaacetic acid (DTPA-ITC, Macrocyclics, Dallas, TX) was added to triskelia (5 mg/ml) in 0.1 M HEPES buffer (pH 8.5) and incubated for 8 hours at 4°C. Chelator was conjugated to protein through lysine residues [40]. Protein was then washed 6 times in 0.1 M ammonium acetate (pH 6) by using Amicon-Ultra-4 with 100 kDa MWCO (Millipore, Billerica, MA) according to the published method [41]. Protein concentration was determined by Bradford protein assay (Bio-Rad, Hercules, CA).

#### Cages

A 15-fold molar excess of DTPA-ITC was added to clathrin triskelia (5 mg/ml) in 0.1 M HEPES buffer (pH 8.5) and incubated for 8 hours at 4°C. Clathrin cages were assembled by mixing modified clathrin triskelia and AP-2 proteins at a ratio of 3∶1 (v/v) according to the standard method [42]. The mixture was dialyzed against MES buffer (50 mM MES Na, pH 6.5, 100 mM NaCl, 2 mM DTT) two times for 12 hours at 4°C, and any unconjugated chelator was separated from nanoplatforms. The relatively high NaCl concentration in the MES buffer was used to facilitate formation of D6 barrel CCs [42]. Aggregated protein was removed by centrifugation in an Eppendorf centrifuge at 15,000 rpm at 4°C for 10 min. Assembled cages were separated from unassembled triskelia by high-speed centrifugation at 60,000 rpm in a TLA-100.4 rotor (Beckman Coulter, US) at 4°C for 12 min, and then resuspended in MES buffer (20 mM MES Na, pH 6.2, 2 mM DTT) to a final concentration of 2.28 mg/ml. Protein concentration was determined by Bradford protein assay (Bio-Rad, Hercules, CA).

#### Arsenazo assay

Ligand (DTPA-ITC) to protein molar ratio was determined by using a spectrophotometric method [43] that was based on reaction between DTPA-ITC-protein conjugate and yttrium (Y^3+^) complex of arsenazo III. Arsenazo III is a highly sensitive colorimetric reagent for yttrium and other metal ions. A 500 ml stock solution of the Y^3+^ -arsenazo III complex contained: 5 µM arsenazo III, 1.6 µM Y^3+^ and 0.15 M sodium acetate buffer (pH 4). From 10 to 60 µL of 0.123 mM DTPA-ITC were added serially to the cuvette that contained Y^3+^ -arsenazo III complex. Absorbance values were measured at 652 nm by using the Spectronic GENESYS 10 Bio spectrophotometer (Thermo Electron Corp., Madison, WI) and a calibration plot constructed. Then, from 20 to 80 µL of DTPA-ITC-protein conjugate were added to the Y^3+^ - arsenazo III complex, and absorbance values were recorded after 10–15 minutes at 652 nm. Unknown concentrations of DTPA-ITC were calculated by using a calibration plot and the following expression:
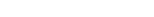
where x represents an unknown concentration of DTPA-ITC, and y is the sample absorbance at 652 nm. After correction for protein dilution, ligand to protein molar ratio was determined. These experiments were done in triplicate.

**Figure 1:**
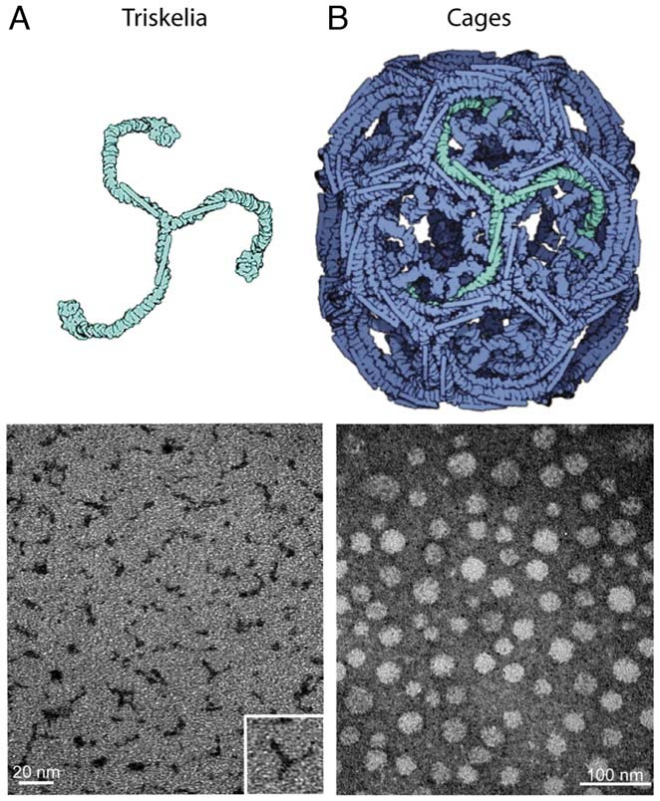
Structure of nanoplatforms. (**A**) The first diagram represents a three-legged clathrin triskelion (light green). Transmission electron microscopy (TEM) image shows clathrin triskelia with attached Gd-DTPA-ITC negatively stained with 1% uranyl acetate. (**B**) The second diagram represents clathrin cage lattice (blue) self-assembled from clathrin triskelia. The TEM image shows clathrin cages with attached Gd-DTPA-ITC negatively stained with 1% uranyl acetate. Clathrin cages formed hexagonal barrels with D6 symmetry.

**Figure 2:**
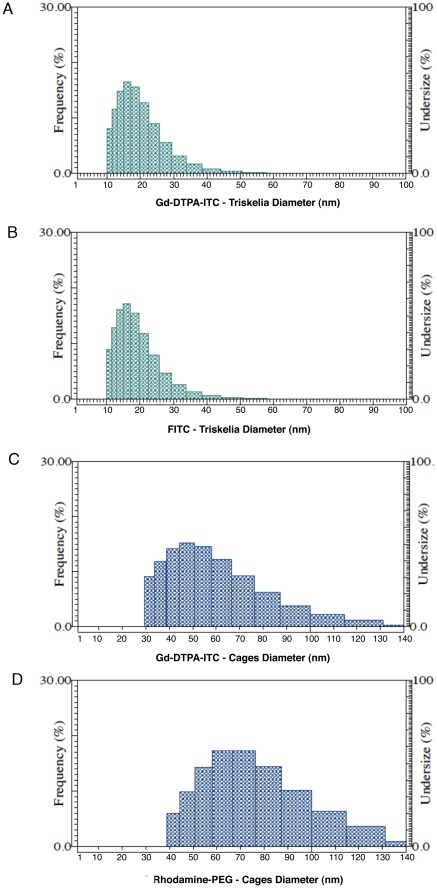
Size of nanoplatforms. Dynamic light scattering (DLS) measurements indicated the mean hydrodynamic radius of (**A**) clathrin triskelia with Gd-DTPA contrast agents was 18.5±6.5 nm and of (**B**) fluorescent FITC-clathrin triskelia was 17.8±6.2 nm. The mean hydrodynamic radius of (**C**) Gd-DTPA-clathrin cages was 55.1±19.7 nm, and of (**D**) fluorescent rhodamine-PEG-clathrin cages was 71.6±21.1 nm.

### Contrast Agent Preparation

Modifications of proteins with Gd-chelates are often performed in two different buffers [41]. Protein chelation is often performed in basic buffers, while metallation with gadolinium is performed in acidic buffers.

#### Cages

Cage-DTPA-ITC nanoplatforms were prepared as previously described. Finally, 0.9 equivalents of Gd-chloride (0.9∶1 molar ratio Gd: DTPA-ITC) were added to a cage-DTPA-ITC mixture (2.28 mg/ml of protein) in 20 mM MES buffer (pH 6.2). After 2 hours, an aliquot was assayed for free Gd^3+^ content using Arsenazo III [44]. Briefly, a 10 µL sample is diluted into 1 ml of 20 µM arsenazo III and analyzed spectrophotometrically. The mixture was then dialyzed against a 500-fold volume excess of MES buffer (20 mM MES Na, pH 6.2, 2 mM DTT) for 12 hours at 4°C, and protein concentration was determined by Bradford protein assay (Bio-Rad, Hercules, CA).

#### Triskelia

To avoid modifying triskelia in acidic buffers, Gd-chelates were prepared separately in ammonium acetate buffer (pH 5.5). Acidic buffers are optimal for cages, but not for triskelia, because triskelia can assemble into polyhedral cages at low pH (<6.5) [45]. Based on spectrophotometric results, 81-fold molar excess of DTPA-ITC (over the amount of triskelia) was solubilised in 100 mM ammonium acetate and pH adjusted to pH 5.5 with acetic acid. Then, 0.9 equivalents of gadolinium chloride were added and reaction incubated at 37°C for 2 hours [46]. An aliquot of gadolinium-DTPA-ITC was assayed for free gadolinium content using arsenazo III [44]. Finally, clathrin triskelia in concentration of about 5 mg/ml in 0.1 M HEPES buffer (pH 8.5) were mixed with prepared Gd-DTPA-ITC for 8 hours at 4°C.

**Figure 3:**
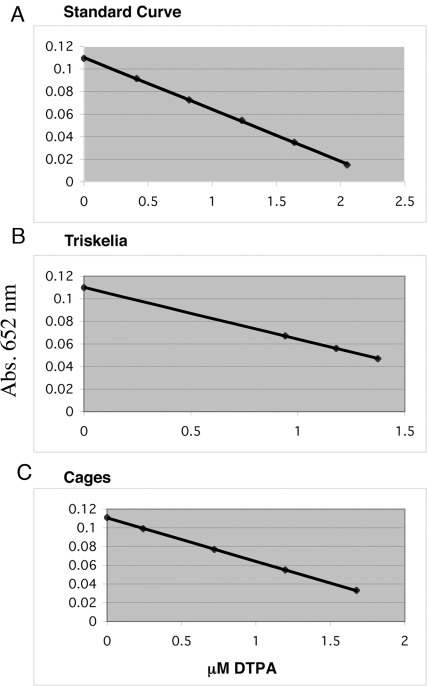
Spectrophotometric method for the determination of a DTPA Ligand. (**A**) Linear relationship between the absorbance of the yttrium complex of arsenazo III at 652 nm and the molarity of DTPA-ITC (R^2^ = 0.999). (**B**) Relationship between the absorbance (A = 652 nm) and the concentration of DTPA-ITC during a sample titration of the yttrium complex of arsenazo III with DTPA-ITC-clathrin triskelia. The mean Ligand (DTPA-ITC)/Protein (Clathrin Heavy Chain) molar ratio was 27.04±4.8: 1. (**C**) The mean Ligand (DTPA-ITC)/Protein (Clathrin Heavy Chain) molar ratio was 4.2±1.04: 1 during a sample titration of the yttrium complex of arsenazo III with DTPA-ITC-clathrin cages.

Unconjugated ligand was separated from the nanoplatforms by dialysis (two times) against a 500-fold volume excess of phosphate buffered saline (PBS) (pH 7.4) at 4°C for 24 hr. Protein concentration was determined by Bradford protein assay (Bio-Rad, Hercules, CA).

### Nanoparticle Characterization

#### Gel analyses

Sodium dodecyl sulfate-polyacrylamide gel electrophoresis (SDS–PAGE) was performed on a Mini-Protean apparatus (Bio-Rad, Hercules, CA). Visualization of protein bands was accomplished by Coomassie Brilliant Blue staining (Bio-Rad, Hercules, CA). Commercially available standards (Bio-Rad, Hercules, CA), and NIH ImageJ software (http://rsb.info.nih.gov/ij) with MolWt macro (http://www.phase-hl.com/imagej.htm) were used for the estimation of molecular weights.

**Figure 4:**
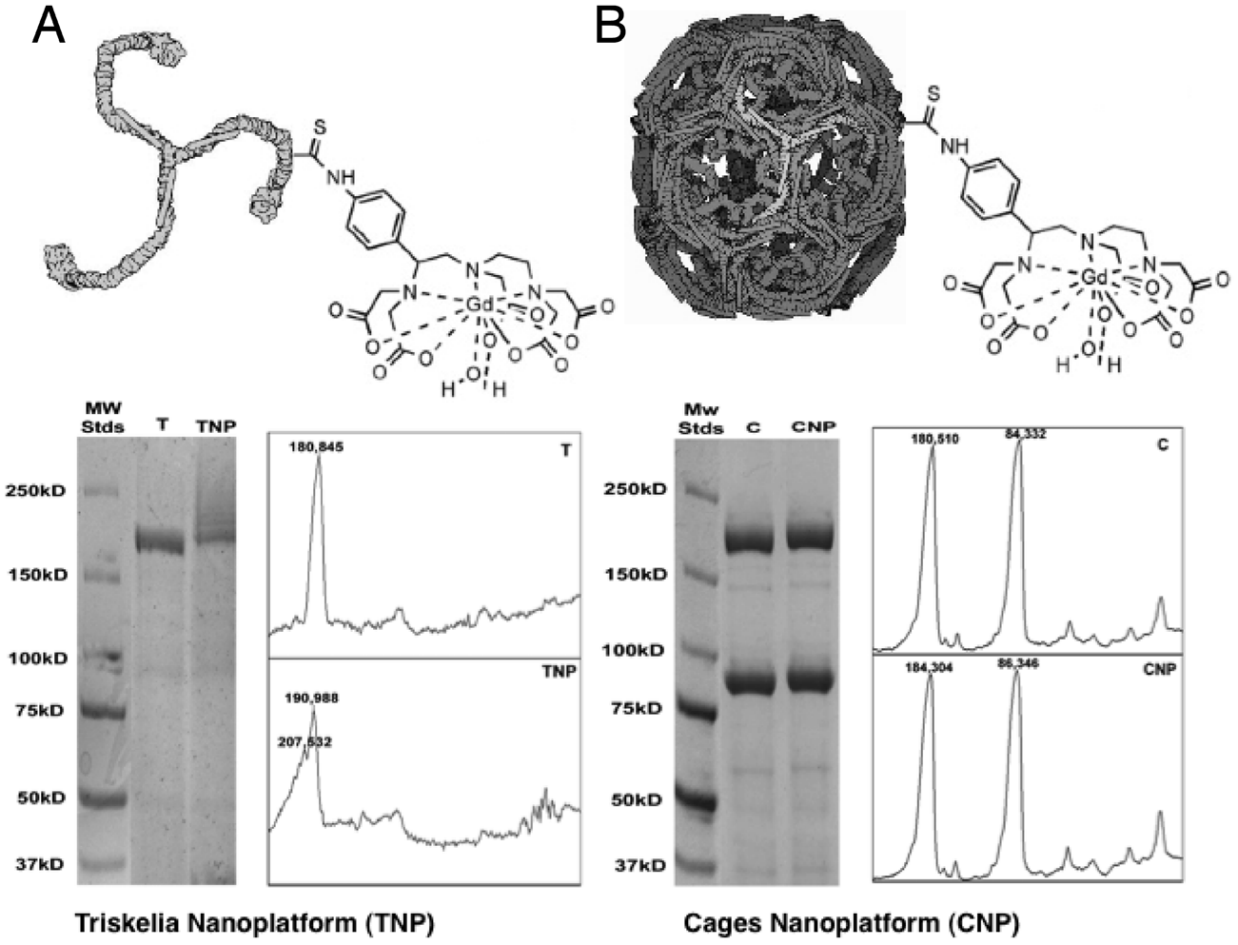
SDS-PAGE of the modified Clathrin nanoplatforms. (**A**) Clathrin triskelion Gd-contrast agent nanoplatform: Line 1. Standards; Line 2. Unmodified Clathrin triskelia; Line 3. Clathrin triskelia with attached Gd-DTPA-ITC. SDS-PAGE analyses show that modified clathrin heavy chain (CHC) bands in triskelia coincide with two molecular weight markers (190,988 kDa and 207, 532 kDa). Molecular weight of the triskelia CHCs increased by 10,143 and 26,687 Da. Thus, between 12.57 and 33.06 molecules of the Gd-DTPA-ITC were attached to the CHC. (**B**) Clathrin cage Gd-contrast agent nanoplatform: Line 1. Standards; Line 2. Unmodified Clathrin cages; Line 3. Clathrin cages with attached Gd-DTPA-ITC. SDS-PAGE analyses show that modified CHC bands in cages coincide with the molecular weight marker of 184,304 kDa. Molecular weight of the cage CHC increased by 3,794 Da, indicating that 4.7 molecules of the Gd-DTPA-ITC were attached to each CHC. Abbreviations: T = triskelia, C = cages, TNP =  triskelia nanoplatform, CNP = cages nanoplatform.

#### Transmission Electron Microscopy (TEM)

Analysis of nanoparticle size and structure was performed on a Jeol 1200 EX electron microscope (Jeol, Tokyo, Japan). About 5 µL of protein solution (0.05 mg/ml) was applied to carbon-coated copper grids for 3 minutes. The grids were rinsed with ddH_2_O, exposed to 5 µL of 1% solution of uranyl acetate (UA), and dried before imaging.

#### Dynamic Light Scattering (DLS)

DLS was performed using a LB-550 (Horiba, Kyoto, Japan) to confirm size and uniformity of nanoparticles.

#### T_1_ Relaxivity

In vitro relaxivity (r_1_) of Gadolinium-DTPA-ITC-nanoparticles was established using a 0.47 T Bruker Minispec NMR system (Bruker, Billerica, MA) at 40°C. The longitudinal relaxation rate (R_1_ = 1/T_1_) was determined from 20 experimental time points generated by an inversion recovery pulse sequence. Longitudinal relaxivity (r_1_) was calculated from the slope of linear least squares fit of 1/T_1_ as a function of Gd^3+^ concentration for different protein concentrations. Triskelia nanoplatforms were in PBS buffer (pH 7.4), the relaxation rate of the PBS buffer was (R_PBS_ = 0.2317), and protein concentration was from 717.64 nmol/L to 5,741.16 nmol/L. Cage nanoplatforms were in MES buffer (pH 6.2, R_MES_  = 0.2477), and protein concentration was from 195.72 nmol/L to 1,565.77 nmol/L.

#### Mineralization monitored by relaxometry

The gadolinium concentration of nanoparticle solutions was measured by a relaxometric procedure according to the standard method [47]. These experiments were performed in triplicate. Briefly, a volume of 750 µL of each solution was added to 750 µL of 70% HNO_3_ directly into a glass ampoule. After gentle centrifugation (1500 rpm, 3 min) ampoules were sealed and heated at 120°C for 5 days to ensure that all Gd^3+^ was solubilised as free aqua ion. Then the water proton T_1_ of these solutions was measured at 20 MHz and 40°C, and Gd^3+^ concentration in starting solutions determined from a standard curve obtained using standard GdCl_3_ solutions (0.0125–0.4 mM), and by using the following expression:
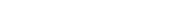
where r_1_ is the relaxivity (mM^−1^s^−1^) of the aqua ion under identical standard experimental conditions, R_1_* (s^−1^) is the relaxation rate of the sample, and R_1B_ (s^−1^) is the relaxation rate of the solution.

### Fluorescent Studies

Brain distribution of clathrin-nanoparticles was assessed in rats using fluorescent analysis of nanoparticles carrying fluorescein-isothiocyanate (FITC, Pierce, Rockford, IL) and rhodamine-PEGs (JenKem, Allen, TX) following intranasal, intraperitoneal, and intravenous administration. FITC labels were conjugated to triskelia using lysine residues [46]. Rhodamine-PEGs were conjugated to clathrin cages using cysteine residues [48]. Florescent-tag to protein molar ratio was determined by spectrophotometric and SDS-PAGE analyses. Dynamic light scattering was performed using a LB-550 (Horiba, Kyoto, Japan) to confirm size and uniformity of nanoparticles.

Male Sprague-Dawley (SD) rats (250 g–300 g) were anesthetized with ketamine/xylazine (80/20 mg/kg). A volume of 70 µL of clathrin nanoparticle PBS solution (33.3 µg of protein) was delivered in nose drops (5 µL per drop) over a 30-minute period. 124 µg of protein in 250 µL of PBS was used for intravenous and intraperitoneal administrations. Animals (n = 4 per time point) were sacrificed and perfused at 30, 60 and 90 minutes following nanoparticle administration. Control animals (n = 2) that did not receive any nanoparticles were sacrificed and perfused before the experiments. Animals were perfused transcardially with saline followed by 4% paraformaldehyde. Brains were removed, post-fixed and cryoprotected in three changes of 30% sucrose. Coronal tissue sections (35 µm) were then cut throughout the entire brain using a microtome. Sections were mounted, coverslipped, and examined using a Zeis Axio Scope A1 photomicroscope (Zeiss, Thornwood, NY).

**Figure 5:**
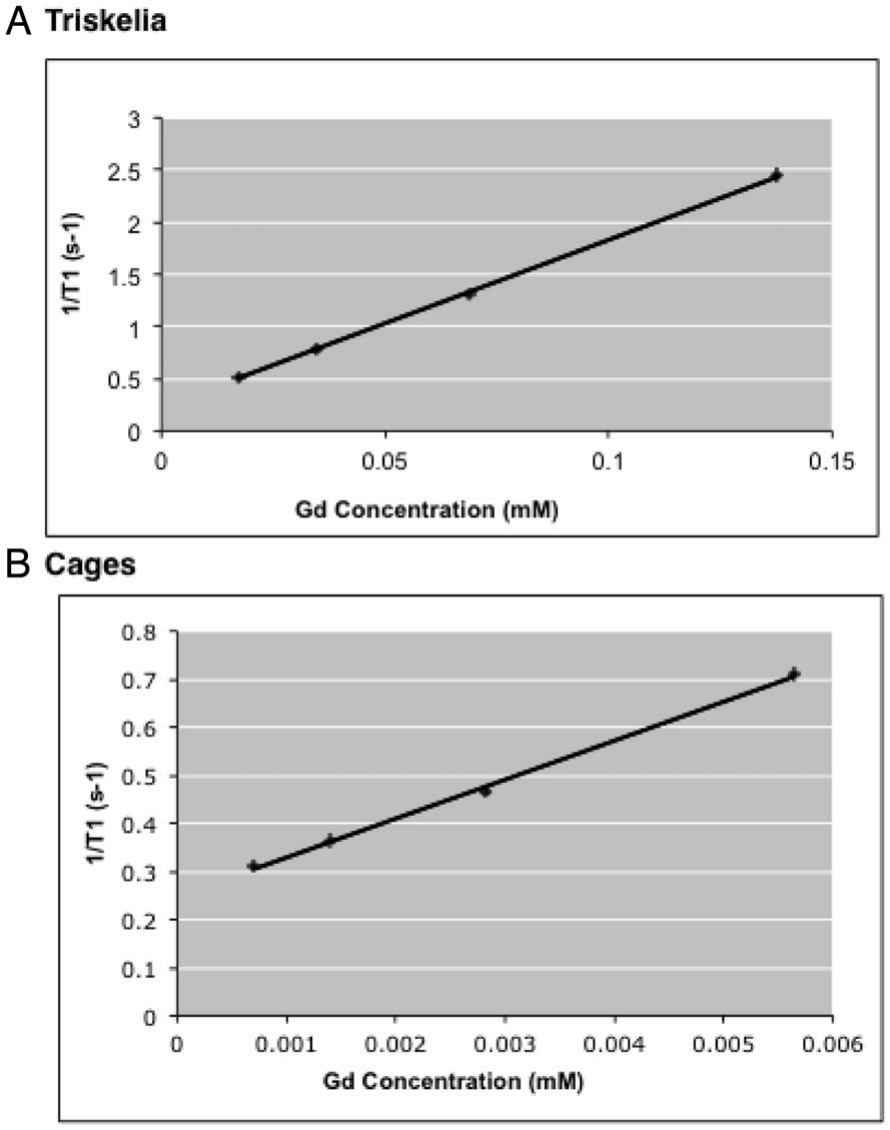
T_1_ Relaxivity of the modified Clathrin nanoplatforms at 0.47 T. (**A**) Solid line (R^2^ = 0.9996) represents a linear relationship between the relaxivity rate of the modified clathrin triskelia and Gd molarity. Triskelia nanoplatforms had ionic relaxivity of 16 mM^−1^s^−1^. Molecular relaxivity was 1,166 mM^−1^s^−1^. (**B**) Linear relationship (R^2^ = 0.9977) between the relaxivity rate of the modified clathrin cages and the Gd molarity. Cages nanoplatforms had ionic relaxivity of 81 mM^−1^s^−1^. Molecular relaxivity was 31,512 mM^−1^s^−1^.

## Results

### Structure of Nanoplatforms

Two different Gd-transporting nanoplatforms were developed. The first utilized a clathrin triskelion (three-legged) protein complex composed of a trimer of clathrin heavy chains (CHC), each bound to a single clathrin light chain (CLC) (Fig.1A). The second Gd-nanoplatform was based on a clathrin-cage self-assembled from clathrin triskelia (Fig. 1B). Electron microscopy showed a large proportion of conjugated Gd-DTPA-clathrin triskelia (Fig. 1A), and also of conjugated Gd-DTPA-clathrin cages (Fig.1B). The majority of Gd-DTPA-CCs had D6 symmetry, formed hexagonal barrels, and had 36 clathrin triskelia, comprising 108 heavy chains and 108 light chains.

**Figure 6:**
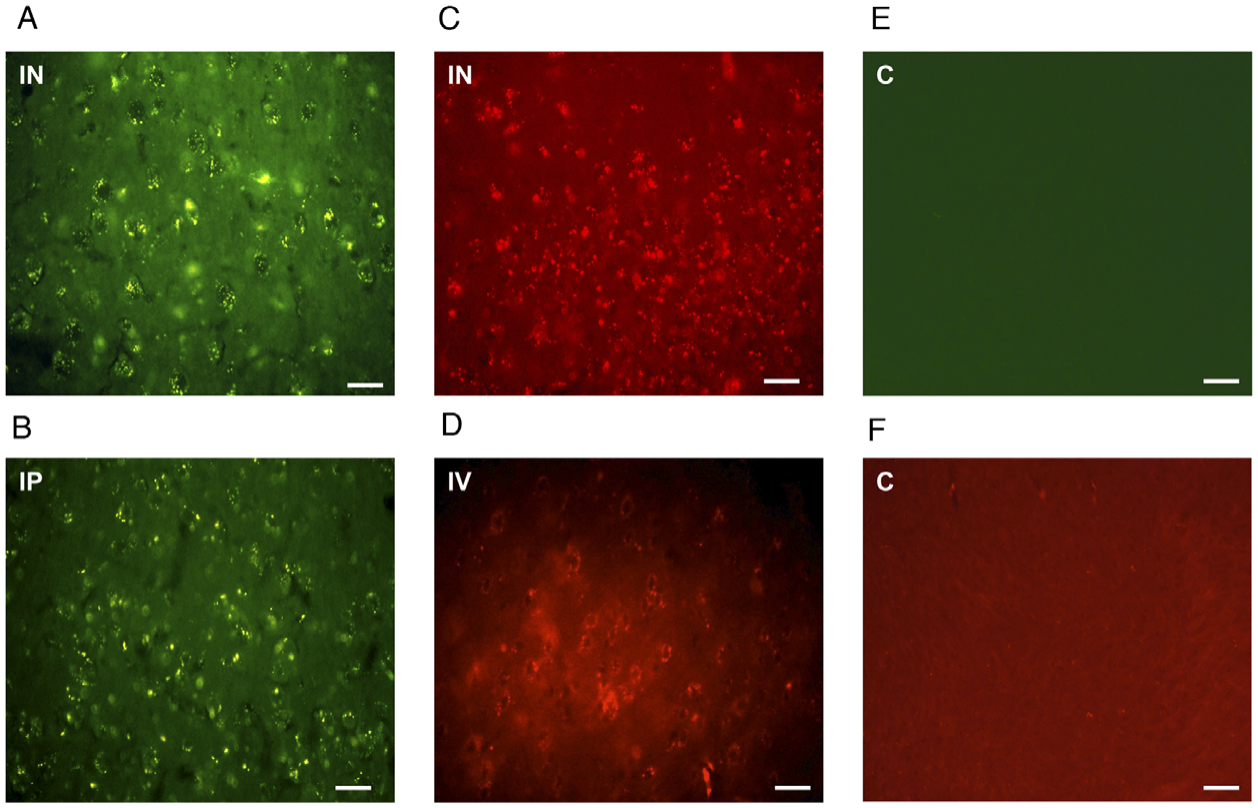
Delivery of Nanoplatforms across the Blood Brain Barrier to the anterior rat brain. Ninety minutes after (**A**) intranasal (IN) and (**B**) intraperitoneal (IP) administration FITC-labeled clathrin-triskelia (green) were identified in all anterior brain regions including the corpus striatum in rats. Also, rhodamine-PEG labeled clathrin cages (red) were identified in the striatum 90 minutes after (**C**) intranasal (IN) and (**D**) intravenous (IV) delivery in rats. Images (**E**) and (**F**) of control (C) animals do not show any fluorescent patterns in the corpus striatum. The scale bar is 100 µm.

**Figure 7:**
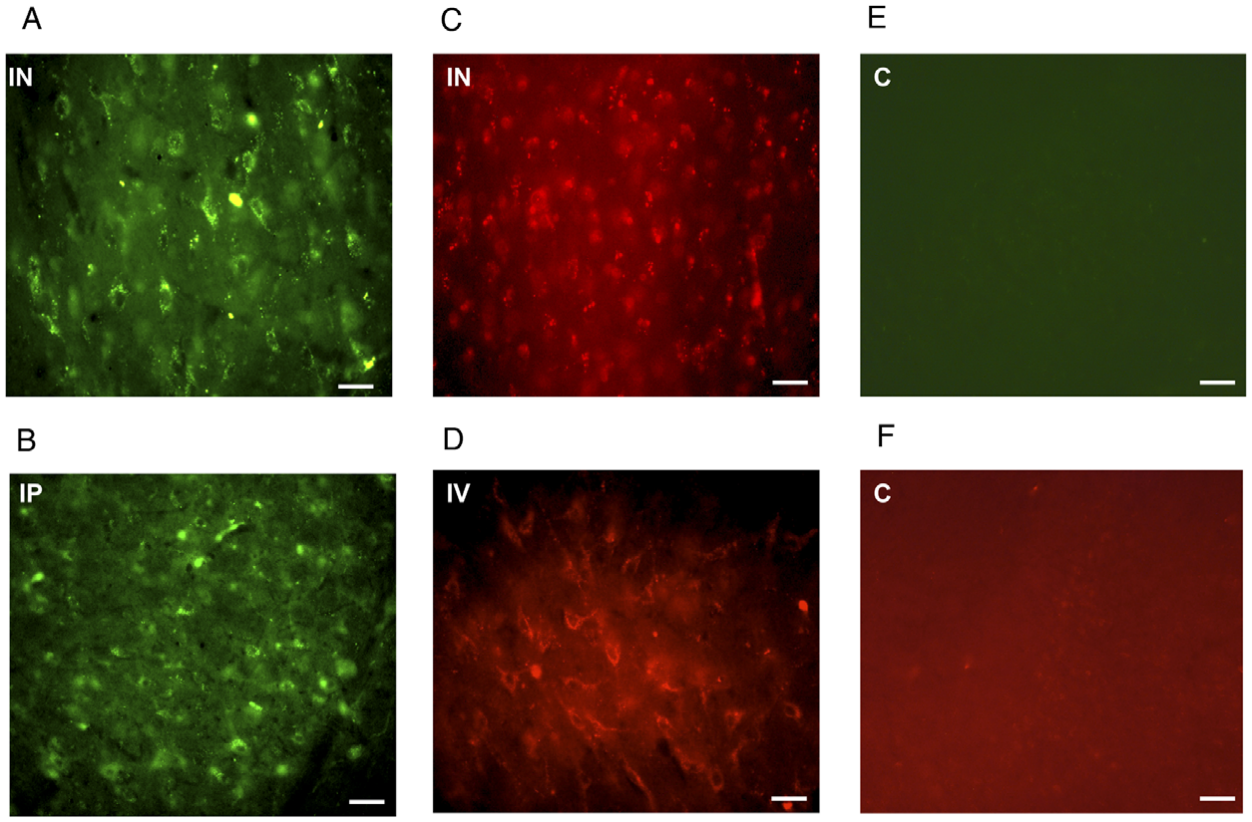
Delivery of Nanoplatforms across the Blood Brain Barrier to the posterior rat brain. Ninety minutes after (**A**) intranasal (IN) and (**B**) intraperitoneal (IP) administration FITC-labeled clathrin-triskelia (green) were identified in all posterior brain regions, including the substantia nigra in rats. Also, rhodamine-PEG labeled clathrin cages (red) were identified in the substantia nigra 90 minutes after (**C**) intranasal (IN) and (**D**) intravenous (IV) delivery in rats. Images (**E**) and (**F**) of control (C) animals do not show any fluorescent patterns in the substantia nigra. The scale bar is 100 µm.

### Size of Nanoplatforms

The mean hydrodynamic radius of clathrin triskelia with attached Gd-DTPA was 18.5±6.5 nm (Figure 2A). Previous DLS studies of clathrin triskelia in solution also reported a Stokes radius of 17 to 18 nm [49], [50]. DLS instruments use spherical models to estimate particle sizes. However, a triskelion is not a spherical particle. A single triskelion has three legs that are bent, puckered, and positioned differently in 3-dimensional space. Electron microscopy has shown that triskelion legs can vary from 35 to 62 nm in total length after straightening [51], [52]. High-resolution atomic force microscopy also confirmed that the legs are flexible along their entire length [53]. Thus, there is variability in the measurements of triskelion size.

The mean hydrodynamic radius of Gd-DTPA-CCs was 55.1±19.7 nm (Figure 2C), which is consistent with EM data for clathrin barrels with D6 symmetry [45]. Thus, there is a slight overlap in the sizes of triskelia and cages.

The mean hydrodynamic radius of FITC-clathrin triskelia was 17.8±6.2 nm (Figure 2B). Thus, FITC-triskelia were similar in size to the GD-DTPA-labeled triskelia. The mean hydrodynamic radius of the rhodamine-PEG-clathrin cages was 71.6±21.1 nm (Figure 2D). Rhodamine-PEG-cages were about 16 nm larger than Gd-labeled cages, because rhodamine-PEGs (MW 3,867 Da, JenKen Tech., Allen TX) were about 16 nm in size.

### Chelate Ligand to Clathrin Protein Molar Ratio

A chelating agent (DTPA-ITC) was attached to clathrin protein, and chelate to protein molar ratio (L/P) determined. Standard spectrophotometric methods were used (Pippin et al. 1992 [43]) based on the reaction between DTPA-ITC ligand protein conjugate and an yttrium (III) complex of arsenazo III. Figure 3A shows dependence of absorbance at 652 nm on DTPA-ITC molarity. Linearity of data demonstrates that Beer’s law was observed over the concentration range of 0−2.0 µM of DTPA-ITC. Absorbance at 652 nm was determined for 20–80 µl solutions of DTPA-ITC-Clathrin-triskelia (Fig. 3B) and DTPA-ITC-Clathrin-cage conjugates (Fig. 3C). Protein concentration was determined by Bradford protein assay. The mean DTPA-ITC: Clathrin Heavy Chain molar ratio was 27.04±4.8: 1 for triskelia, and 4.2±1.04: 1 for cages.

SDS-PAGE image analyses showed the molecular weight of modified clathrin heavy chain (CHC) in the triskelia-based nanoplatform had changed from 184,845 to between 190,988 Da and 207,532 Da (Fig. 4A), whereas CHCs in the CC-based nanoplatform had changed from 180,510 Da to 184,304 Da (Fig. 4B). These data are consistent with spectrophotometric data.

Rat CHC has 1675 amino acid residues, of which 97 are lysine residues, but only some are available for conjugation with DTPA-ITC. Molecular weight of the cage CHC increased by 3,794 Da, indicating that 4.7 molecules of Gd-DTPA-ITC were attached to each CHC.

After the modifications of CHC with GD-DTPA ITC in the triskelia nanoplatform (TNP) we found 2 peaks that indicated different molecular weights (190,988 Da and 207,532 Da). Thus, molecular weight of triskelia CHCs increased by 10,143 Da (from 180,845 Da to 190,988 Da), and by 26,687 Da (from 180,845 Da to 207,532 Da). Thus, between 12.57 and 33.06 molecules of Gd-DTPA-ITC were attached to each CHC.

Fewer Gd-DTPA-ITC molecules were attached to CHCs in the cage nanoplatform, because of a solubility problem. Cages that averaged greater than 7 modifications per CHC were found to easily precipitate, although diluting the sample could attenuate precipitation. Ultimately, poor stability of these highly decorated particles limited their characterization and subsequent Gd^3+^ metallation, whereas cages with fewer DTPA-ITC molecules were stable and not prone to aggregation.

**Table 1:** MRI Nanoplatforms (at 20 MHz).

	r_1_(mM^−1^s^−1^)	References
Gd-TREN-bis-HOPO-TAM-CO_2_H	7.3	Pierre et al. 2006 [70]
***Clathrin triskelia-Gd-DTPA-ITC***	**16**	*This work*
Gadomer 17	16.5	Nicolle et al. 2002 [71]
PAMAM-G4-Dendrimer-DOTA-Gd	31.2	Jaszberenyi et al. 2007 [72]
PAMAM-G10-Dendrimer-DOTA-Gd	36	Bryant et al. 1999 [61]
MS2-TREN-bis-HOPO-TAM	38.4	Datta et al. 2008 [47]
Apoferritin-HPDO3A	80	Aime et al. 2002 [55]
***Clathrin cages-Gd-DTPA-ITC***	**81**	*This work*

### Relaxivity and Gadolinium Content Measurements

Gadolinium concentrations and T_1_ relaxivities were determined for triskelia and cages. Gadolinium concentrations were measured by relaxometry [47] and spectrophotometric methods [44]. Spectrophotometric results indicated that 100% of added gadolinium was chelated by DTPA-ITC. The Gd to DTPA-ITC molar ratio was 0.9∶1. Nuclear Magnetic Resonance (NMR) results confirmed spectrophotometric results for clathrin triskelia. The Gd^3+^ concentration in 750 µl of triskelia conjugate was 0.0689 mM according to spectrophotometric methods, and 0.0693 mM according to NMR methods. The Gd^3+^ concentration in 750 µl of cage conjugate was 0.0028 mM according to spectrophotometric methods, and could not be detected by NMR methods. Gd^3+^ concentration in cage conjugate was below the 0.01 mM detection limit of NMR [54], [55].

Relaxivities for each sample were calculated using T_1_ data and spectrophotometrically determined gadolinium concentrations. At 0.47 T, Gd-DTPA-ITC-triskelia displayed a relaxivity of 16 mM^−1^s^−1^per gadolinium ion (Fig. 5A) and 1,166 mM^−1^s^−1^per particle. However, Gd-DTPA-ITC-cages displayed a relaxivity of 81 mM^−1^s^−1 ^per gadolinium ion (Fig. 5B), and 31,512 mM^−1^s^−1 ^per particle. Thus, triskelia exhibited 4 times higher ionic relaxivity, and 291.5 times higher molecular relaxivity compared to Gd-DTPA. Cages displayed 20 times higher relaxivity per gadolinium ion than expected for a corresponding amount of Gd-DTPA. Finally, clathrin cages had over 7,878-fold greater molecular relaxivity than traditional Gd-MR contrast agents, like gadopentetate dimeglumine. Diluted samples showed slightly higher ionic relaxivities (22 mM^−1^s^−1^ for triskelia, and 97 mM^−1^s^−1^ for cages).

### Fluorescent Clathrin Nanoplatforms In-Vivo

In order to determine whether clathrin nanoplatforms could cross or bypass the BBB in rats, triskelia and cages were modified with fluorescent tags. Fluorescent FITC labels were conjugated to triskelia through reactive lysine residues using a Pierce FITC Labeling Kit (Figures 6–7). On average, 27.48 molecules of FITC were attached per triskelion complex, and the mean hydrodynamic radius of FITC-clathrin triskelia was 17.8±6.2 nm (Fig. 2B). Fluorescent triskelia-nanoparticles were administered intranasally (i.n.) and intraperitoneally (i.p.) in male Sprague-Dawley (SD) rats (250 g–300 g) at 30, 60, and 90 minutes time points. Ninety minutes after i.n. and i.p. administration, FITC-labeled clathrin-triskelia were identified in all rat brain regions examined, including dopamine related areas (Figures 6, 7). Particles were also present in brain regions 30 and 60 minutes post i.n. and i.p. administration. Thus, clathrin triskelia successfully bypassed the BBB when delivered intra-nasally, and/or crossed the BBB when delivered intra-peritoneally, and were widely distributed throughout the brain.

Next, rhodamine-PEGs were conjugated to clathrin cages through reactive cysteine residues. 4.76 rhodamine-PEG molecules were attached to each CHC of the cage, and the mean hydrodynamic radius of the rhodamine-PEG-clathrin cages was 71.6±21.1 nm (Fig. 2D). Nanoparticles were administered to rats through their tail veins or intranasally. Rhodamine fluorescence was observed in all brain areas examined, including dopamine-rich regions 90 minutes after i.v. and i.n. administration (Figures 6, 7).

## Discussion

Two different Gd-nanoplatform methods were developed to show feasibility of Clathrin protein-based imaging techniques. The first method utilized a clathrin mono-unit (triskelion) with a radius of 18.5 nm. This measure compares well with other DLS studies of clathrin triskelion showing a Stokes radius of 17 to 18 nm [50]. An individual triskelion consists of three 190 kDa (1,675-residue) heavy chains, each bearing a single 25 kDa light chain [31]. A triskelion has an apparent native ability to enter cells (e.g., neurons [37]). Thus, triskelia nanoplatforms may offer significant potential in support of imaging of intracellular molecular markers and cell signaling pathways, for cellular tracking/imaging, and for intracellular delivery of drugs, genes and/or antisense oligonucleotides.

The second method utilized a Gd-nanoplatform (size, 55.1 nm) based on clathrin cages composed of self-assembled triskelia. Triskelia legs create a lattice of hexagonal and pentagonal faces, and cages exhibit a range of three-dimensional designs [42]. The most frequently formed cage-like structures are built from 28, 36, and 60 triskelia, which, respectively, are a ‘mini-coat’ with tetrahedral symmetry, a ‘hexagonal barrel’ with D6 symmetry, and a ‘soccer ball’ with icosahedral symmetry. Because of a clathrin cage’s native ability to encapsulate and protect a wide range of molecular structures (e.g., hormones, peptides, proteins, antibodies, neurotransmitters) [30], cage nanoplatforms could be utilized to deliver different ligands and/or drugs to specific sites of action. Also, clathrin coats can be assembled on liposomes to form clathrin-coated vesicles (CCV’s) [33]. Using vesicles could afford another nano-transport cargo technique. Further, using free-floating CA-complexes within assembled cages could present another interesting target of opportunity [56].

The study goal was to create a method that would yield stable, Gd-nanoplatforms that could provide enhanced CA imaging performance. Chelate ligand (DTPA-ITC) was attached to clathrin protein, and chelate to clathrin protein molar ratio was determined by using standard spectrophotometric methods [43]. Optimal DTPA-ITC loading for a single clathrin triskelion was 81, and 432 for a complete clathrin cage. Complete saturation of all binding sites may result in a large number of metals attached to a single nanoparticle complex, which is important for different imaging and therapeutic modalities. However, clathrin cages with a large number of chelating agents tended to precipitate, which is consistent with some virus nanoparticle reports [57]. Encapsulating metal chelates, or attaching metal chelates to a clathrin cage’s interior could prevent precipitation. Similar strategies have been used with apoferritin [55], silicon particles [58] and viruses [57].

The smaller Gd-DTPA-ITC-triskelion platform displayed a longitudinal relaxivity four times greater than that of the monomeric chelate, and was similar to relaxivities reported for some proteins (e.g., albumin, fibrinogen, IgG) [59], linear polymers (e.g., poly-L-lysine) [60] and generation-5 dendrimers [61] that were covalently bound to Gd-DTPA.

The cage-based CA nanoplatform displayed about 20-fold greater ionic relaxivity than the monomeric chelate. In vitro measurements of relaxivity at clinically relevant field strength demonstrated additional gains from slow tumbling rates of spherical clathrin cages. Observed ionic relaxivity is consistent with relaxivity enhancement due to rotational correlation effects, high local gadolinium concentrations, and relatively fast water exchange inside a cage as reported in previous virus studies [12], [46], [57], [62]. However, nuclear magnetic resonance dispersion (NMDR) studies need to be performed to further clarify relaxivity issues.

Observed values were similar to values reported for apoferritin [55]. Also, ionic relaxivities were higher than those reported for other nanoparticles at 20 MHz [47] (Table 1). PAMAM dendrimers (G = 5, 7, 9, and 10) also display a high ionic relaxivity (from 30 to 36 mM^−1^s^−1^) at 20 MHz [61]. Although the ionic relaxivity did not increase, the total molecular relaxivities increased (from 2,880 mM^−1^s^−1^ to 66,960 mM^−1^s^−1^) from generation-5 to generation-10 dendrimers. Molecular relaxivity was about 2 times higher in generation-10 dendrimers compared to the clathrin cages. However, it may be possible to modify porous clathrin cages to carry a higher number of Gd^+3^ ions inside the protein cage to increase their molecular relaxivity.

Observed values were also lower than those reported for Gd^+3^ ions attached to Calcium binding sites of wild-type or bioengineered cowpea chlorotic mottle virus (CCMV) [202 mM^−1^s^−1^ at 61 MHz [63] and 210 mM^−1^s^−1^ respectively [62]]. However, these sites bind Gd^3+^ too weakly for clinical use. Also, endohedral metallofullerenes [58], [64] with a similar fullerene-like cage structure to clathrin displayed a high ionic relaxivity (from 102 mM^−1^s^−1^ to 200 mM^−1^s^−1^). However, their toxicity is not fully understood.

Some studies have focused on increasing molecular relaxivity per particle by developing larger particles (over 100 nm in size) that can carry a high payload of gadolinium CA. For example, porous polymersomes (ca. 125 nm) encapsulated nearly 44,000 Gd^+3^ per particle, and exhibited molecular relaxivity of 320,000 mM^−1^s^−1^ [65]. Also, paramagnetic liquid perfluorocarbon nanoparticles (ca. 250 nm) with over 90,000 Gd^3+^ per particle exhibited ionic relaxivity of 17.9 mM^−1^s^−1^, and molecular relaxivity of 1,690,000 mM^−1^s^−1^ at 1.5T [9]. Thus, particles 2.5 to 5 times larger then clathrin cages demonstrated lower ionic relaxivity, but showed higher molecular relaxivity because of a higher number of gadolinium CA. However, particles over 100 nm in size have not been shown to cross the BBB [5].

Some studies have created clusters of nanoparticles to increase their molecular relaxivity. For example, gadolinium-conjugated dendrimer nanoclusters (DNCs) were prepared by crosslinking fifth-generation PAMAM dendrimers with crosslinkers (e.g., NHS-PEG-NHS) [66]. Paramagnetic DNCs were about 150 nm in diameter, had an r_1_ relaxivity value of only 12.3 mM^−1^s^−1^ per Gd^3+^, but showed molecular relaxivity of approximately 3,600,000 mM^−1^s^−1^. Clathrin cages can also be crosslinked with PEGs to form nanoclusters, which may increase their molecular relaxivity, but may also limit their ability to cross the BBB [5].

To determine if these novel nanoplatforms could also enable in vivo, noninvasive delivery into the CNS, fluorescent-tagged triskelia and cages were designed, and utility for rat brain imaging pilot-tested. These studies provide first evidence that fluorescent-tagged clathrin nanoplatforms were successfully delivered non-invasively into rat brain. Significantly, both triskelia and cages crossed and/or bypassed the BBB without enhancers or modifications, unlike other nanoparticle types [5]. One limitation is that Gd-CA was not attached to the nanoplatforms. Also, the mechanism of clathrin transport through the BBB is still unknown. Prior studies showed [37] clathrin can be released by neurons and move between them. Animal studies are currently conducted to: clarify a mechanism of entry of clathrin nanoparticles into the CNS; quantify nanoparticles in different organs and tissues; evaluate if these nanoparticles with Gd-CA non-invasively enter the CNS; and determine if they elicit CNS toxicity.

Thus, potent T_1_ Gd-DTPA contrast agents were created using these novel nano-methods. However, a limitation was that nanoparticle characterizations were performed at 20 MHz. It is unclear whether similar relaxivities would be observed at other field strengths. Lower relaxivities were found for T_1_ contrast agents at higher fields [67], [68]. Further studies are needed to find optimal Gd-DTPA positioning (e.g., external vs. internal) and loading for clathrin-nanoplatforms. Some studies showed longitudinal relaxivities increased initially with increasing Gd-DTPA/protein ratios, and reached a plateau at a particular Gd-DTPA/protein ratio [69]. We reported T_1_ measures, but T_2_ measures should also be performed and an *r*_2_/*r*_1_ ratio estimated. In general, *r*_1_ should be as large as possible, and *r*_2_/*r*_1_ ratio should be as close to 1 as possible in order for a nanoparticle to be used as a highly sensitive T_1_ MRI contrast agent. In vitro experiments indicated that a clathrin-based CA could produce as much contrast as currently approved MRI contrast agents, but do so at much lower concentrations, which is important for minimizing Gd-CA toxicity in clinical applications. More important, the addition of ligands or antibodies to the nanoplatform may provide the specificity needed for molecular imaging. Further in-vivo MRI studies are required to determine minimal MRI-visible concentration and test stability, toxicity, biodistribution, and the general feasibility of these new nanoplatforms for MR imaging. Finally, further in vivo studies will show whether this novel nanoplatform can act as a potent, non-invasive transporter into the CNS of Gd-CAs.

### Conclusion

It was herein shown: 1) A new CA method utilizing Gd and Clathrin bio-nanoparticles is feasible; 2) Clathrin protein proved a robust nanoplatform onto which multiple functional motifs could be added through chemical modifications of different amino acid residues; 3) a single clathrin cage can carry hundreds of Gd^3+^ ions and has among the highest ionic relaxivity found for a Gd-DTPA CA; 4) Clathrin nanoplatforms are size-adjustable (18 to 55 nm in size); 5) clathrin cages are relatively stiff molecular structures with large rotational correlation times, resulting in increased relaxivity rates; and 6) fluorescent clathrin cages and triskelia can cross or bypass the BBB without enhancers or modifications, and have potential for non-invasive CNS imaging. These preliminary results should encourage further investigation into this new nanoplatform method for Gd-based imaging.
